# Combined In Silico and Experimental Investigations of Resveratrol Encapsulation by Beta-Cyclodextrin

**DOI:** 10.3390/plants11131678

**Published:** 2022-06-24

**Authors:** Ainara Iskineyeva, Serik Fazylov, Ryszhan Bakirova, Akmaral Sarsenbekova, Irina Pustolaikina, Olzhas Seilkhanov, Aisha A. Alsfouk, Eslam B. Elkaeed, Ibrahim H. Eissa, Ahmed M. Metwaly

**Affiliations:** 1 Saken Seifullin Kazakh Agrotechnical University, Nur-Sultan 010000, Kazakhstan; iskeneeva_aynara@mail.ru; 2 Chemistry Faculty, Karagandy University of the Name of Academician E.A. Buketov, Karaganda 100024, Kazakhstan; chem_akmaral@mail.ru (A.S.); ipustolaikina@gmail.com (I.P.); 3 Karaganda Medical University, Karaganda 100012, Kazakhstan; r.bakirova@qmu.kz; 4 Kokshetau State University, Kokshetau 020000, Kazakhstan; seilkhanov@mail.ru; 5 Department of Pharmaceutical Sciences, College of Pharmacy, Princess Nourah bint Abdulrahman University, P.O. Box 84428, Riyadh 11671, Saudi Arabia; aaalsfouk@pnu.edu.sa; 6 Department of Pharmaceutical Sciences, College of Pharmacy, AlMaarefa University, Riyadh 13713, Saudi Arabia; ikaeed@mcst.edu.sa; 7 Pharmaceutical Medicinal Chemistry & Drug Design Department, Faculty of Pharmacy (Boys), Al-Azhar University, Cairo 11884, Egypt; ibrahimeissa@azhar.edu.eg; 8 Pharmacognosy and Medicinal Plants Department, Faculty of Pharmacy (Boys), Al-Azhar University, Cairo 11884, Egypt; 9 Biopharmaceutical Product Research Department, Genetic Engineering and Biotechnology Research Institute, City of Scientific Research and Technological Applications, Alexandria 21934, Egypt

**Keywords:** cyclodextrins, β-cyclodextrin, vitamin resveratrol, molecular modeling, inclusion complexes, antioxidant activity, clathrate

## Abstract

The results of the computational and the physicochemical studies of the encapsulation of resveratrol with β-cyclodextrin are presented here. At first, the molecular docking experiments predicted good binding. Several MD simulations and MM-PBSA experiments confirmed the reliable binding, showing optimal kinetics and energy. As an application, resveratrol inclusion complexes with β-cyclodextrin were obtained in an aqueous alcohol medium via microwave treatment. The results of thermographic measurements of the obtained clathrates using a differential scanning calorimeter are presented, and the obtained activation energy was calculated using the Ozawa–Flynn–Wall and Friedman methods, as well as nonparametric kinetics. The effect of complexation on the kinetic parameters of thermal destruction of the β-cyclodextrin–resveratrol inclusion complex was considered. The morphology of the surface of the obtained clathrate complexes was described using a scanning electron microscope. The spectral properties of the inclusion complex were characterized by FT-IR, ^1^H, and ^13^С NMR spectroscopic data. The obtained in silico, morphological, thermogravimetric, and spectral results confirmed the formation of the resveratrol–β-cyclodextrin complex. The antioxidant activities of the inclusion complex were determined to be 12.1 μg/mL, compared to 14.3 μg/mL for free resveratrol, indicating an improvement in the bioactivity.

## 1. Introduction

**Figure 1:**
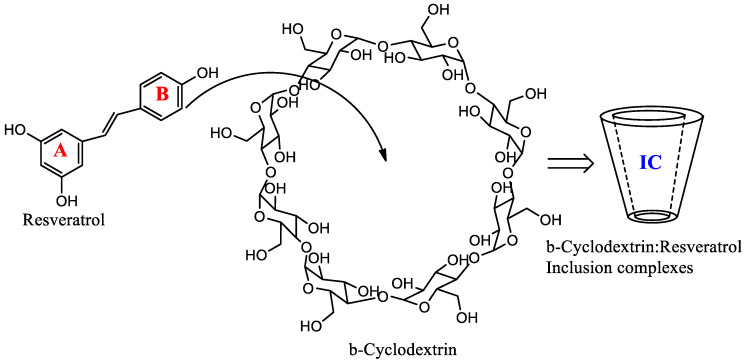
Structural formulae of resveratrol contain aromatic rings A and B, β-cyclodextrin, and inclusion complexes (IC).

Resveratrol, (Figure 1) is a natural stilbene that is produced by various plants as a phytoalexin responding to infection or injury [1]. Extracts containing resveratrol have previously been used in traditional Chinese and Japanese medicine for treating various diseases [2]. Some studies have provided evidence that resveratrol modulates inflammation through TLR-4 attenuation [3], and inhibits platelet activation and aggregation [4]. Resveratrol showed anticancer effects through targeting the mitochondrial- and p53-signaling-dependent apoptosis [5], NF-κB and AP-1 [6], and the p53 tumor-suppressor protein [7], in addition to several preclinical and clinical trials that indicated the anticancer activities of resveratrol [8,9,10,11]. Resveratrol exhibited activity against bacterial and fungal species that are major etiological agents in human skin infections [12,13,14,15]. However, the effectiveness of resveratrol preparations in vivo is still limited due to their low bioavailability, which is a consequence of their low solubility in water and rapid removal from circulation [16]. On the other hand, resveratrol deserves great attention as a food additive that has antioxidant, and antimicrobial properties [17,18,19]. To overcome the poor pharmacokinetics of resveratrol, some trials have been carried out to encapsulate it with cyclodextrins (CDs) to increase its bioavailability, solubility, and stability against light, oxygen, and inorganic salts [20,21]. However, there is still a need to search for new methods to improve its solubility, bioavailability, and oxidation resistance. In this investigation, we tried to study the features of the encapsulation process of resveratrol with β-CD at different ratios (i.e., 1:2 and 1:4), using various in silico (i.e., modeling, MD simulations, and MM-PBSA) and physicochemical methods. The in silico results showed accurate binding, with perfect kinetics and energy. Additionally, the physicochemical studies determined the kinetic characteristics of the thermal decomposition process, namely, the activation energy and the pre-exponential multiplier. These parameters were determined based on the Freeman–Carroll, Sharpe–Wentworth, Ahar, and Coates–Redfern methods, which have good convergence. The dependence of the reaction rate on the temperature in a nitrogen medium has been studied by nonparametric kinetics methods.

## 2. Results and Discussion

### 2.1. Molecular Docking Studies

#### 2.1.1. Investigation of Binding Free Energy and Binding Mode

Discovery Studio was employed to investigate the binding pattern of resveratrol with β-CD. β-CD cavity is O-shaped; it consists of seven glucose units. These units are involved in a hydrogen bond network between the β-CD and resveratrol.

**Figure 2:**
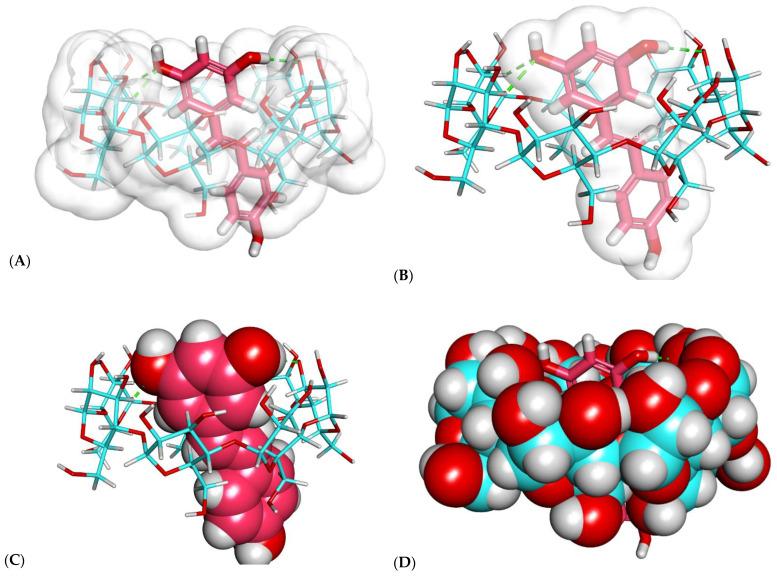
Docked poses of the best-ranked docking score of resveratrol with β-CD (front view): (**A**) resveratrol (sticks) and β-CD (sticks) pointing at β-CD, (**B**) resveratrol (sticks) and β-CD (sticks) pointing at resveratrol, (**C**) resveratrol (CPK) and β-CD (sticks), (**D**) resveratrol (sticks) and β-CD (CPK).

**Figure 3:**
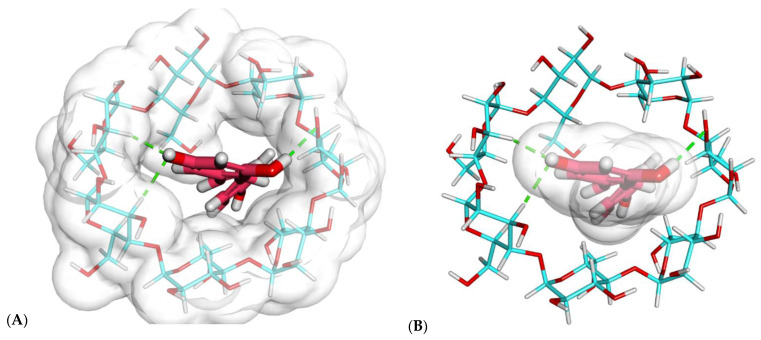

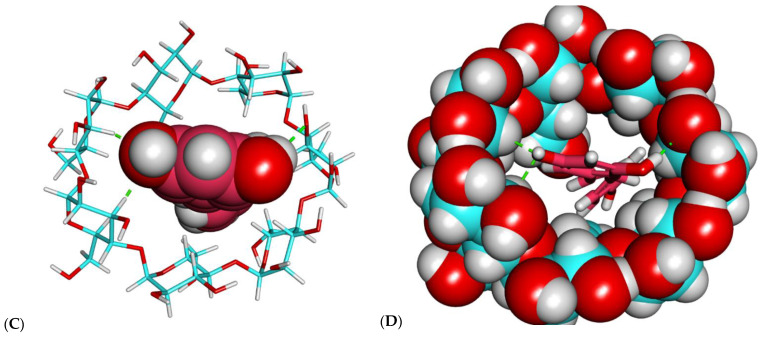
Docked poses of the best-ranked docking score of resveratrol with β-CD (top view): (**A**) resveratrol (sticks) and β-CD (sticks) pointing at β-CD, (**B**) resveratrol (sticks) and β-CD (sticks) pointing at resveratrol, (**C**) resveratrol (CPK) and β-CD (sticks), (**D**) resveratrol (sticks) and β-CD (CPK).

As shown in Figure 2 and Figure 3, resveratrol exhibited a good binding mode with β-CD, with a binding score of −23.25 kcal/mol.

The proposed binding mode of resveratrol with β-CD formed many important interactions. The resorcinol moiety of resveratrol formed three hydrogen bonds with the different OH groups of the glucose subunits.

As shown in Figure 2 and Figure 3, the resveratrol was incorporated correctly inside the β-CD. However, one essential drawback emerged, as the size of resveratrol was greater than the length of β-CD (Figure 2B,C). Consequently, another modeling study of β-CD–resveratrol complexes was performed using the HyperChem 8.0 program (Hypercube Inc., Gainesville, FL, USA) to examine the ability of one β-CD molecule to enclose more than one resveratrol molecule.

#### 2.1.2. Molecular Modeling of β-CD–Resveratrol Complexes with Different Ratios

The mechanisms of encapsulation of resveratrol with β-CD at different ratios were studied by molecular modeling. Molecular modeling of β-CD–resveratrol complexes was performed using the HyperChem 8.0 program. The calculation method was AM1. Initially, the geometry of the objects of study was optimized—the molecular structures of *trans*-resveratrol (E_total_ = −67,293.92194 kcal/mol, μ = 0.3399 Debye) and *cis*-resveratrol (E_total_ = −67,295.77961 kcal/mol, μ = 0.6555 Debye), as well as β-CD (E_total_ = −405,117.9107 kcal/mol, μ = 3.283 Debye). It is known from the literature [16] that resveratrol exists in two biologically active conformations, while in the condensed phase *trans*-resveratrol is more stable than *cis*-resveratrol. The geometric parameters of the optimized structures of the research objects showed the possibility of the formation of a complex between β-CD and resveratrol molecules. The spatial “elongation” of the *trans*-resveratrol molecule does not exclude the possibility of the formation of complexes with cyclodextrins, with the stoichiometry of both 1:1 and 2:1.

**Figure 4:**
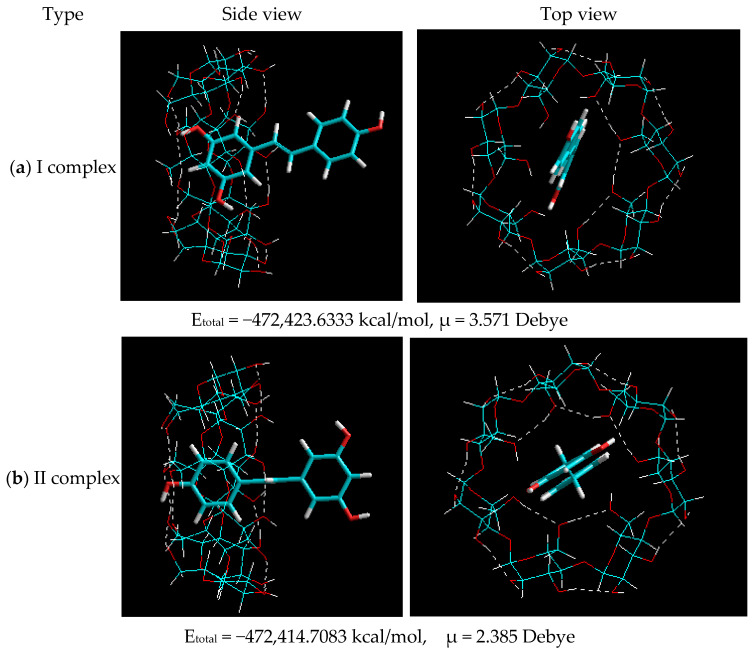
Optimized structure of the type I β-CD–resveratrol complex at a ratio of 1:1 (inclusion of aromatic ring A (**a**) and aromatic ring B (**b**)). Dotted lines show hydrogen bonds.

At the next stage, molecular modeling of *trans*-resveratrol complexes with a β-CD ratio of 1:1 was implemented. The structures of the optimized complexes are shown in Figure 4.

The data presented in Figure 4 demonstrate that based on the interaction of *trans*-resveratrol with β-CD at a ratio of 1:1, the generation of two types of inclusion complex is feasible:

- Type I—as a result of the penetration of the *A* (Figure 4) ring with two hydroxyl groups of *trans*-resveratrol into the β-CD cavity;
- Type II—as a result of the penetration of the *B* (Figure 4) ring with one hydroxyl group of *trans*-resveratrol into the β-CD cavity.

**Figure 5:**
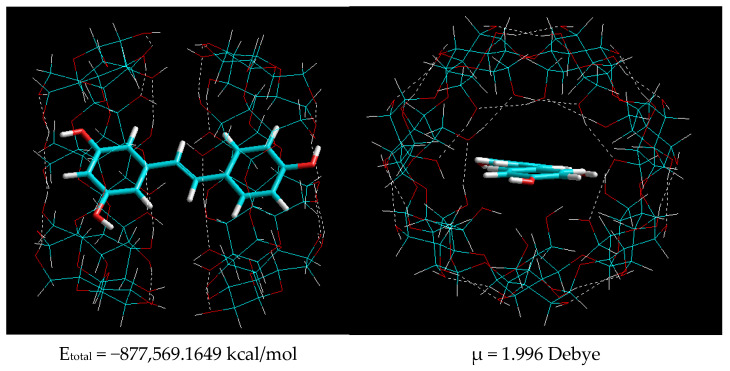
Optimized structure of the type II β-CD–resveratrol complex at a 2:1 ratio.

Comparison of the total energies that resulted from the generation of these two types in Figure 4 shows that the formation of the β-CD–resveratrol inclusion complex by type I at ∆E = 8.925 kcal/mol is thermodynamically more favorable. However, a small difference in energies implies an equal probability of the formation of complexes of types I and II. It should also be noted that the dipole moments of the two types of complexes were practically the same, emphasizing the similarity of their characteristics and stability. Then, molecular modeling of β-CD–resveratrol inclusion complexes was conducted at a ratio of 2:1. Molecular models of β-CD–resveratrol complexes obtained after geometric optimization by the semi-empirical AM1 method at a 2:1 ratio are shown in Figure 5.

The data presented in Figure 5 show that as a result of the complexation of resveratrol with two β-cyclodextrin molecules, the molecule of the former was noticeably “stretched”, while its benzene rings were located at an angle of ~10^o^ to one another. Moreover, the system of hydrogen bonds in the cyclodextrin molecule could be noted in the β-CD–resveratrol inclusion complexes. However, the formation of hydrogen bonds between resveratrol and cyclodextrin molecules was not observed.

It was interesting to observe the change in the total energy after the formation of the β-CD–resveratrol complex. The complexation energy was estimated for all types of complexes as the difference between the sum of the total energies of the molecules of the starting substances and the total energy of the resulting complex, as follows:

ΔE_complex_ = (E_total_ (cyclodextrin) + E_total_ (ligand)) – E_total_ (inclusion complex)

The calculated values of the complex formation energies are presented in Table 1.

**Figure 6:**
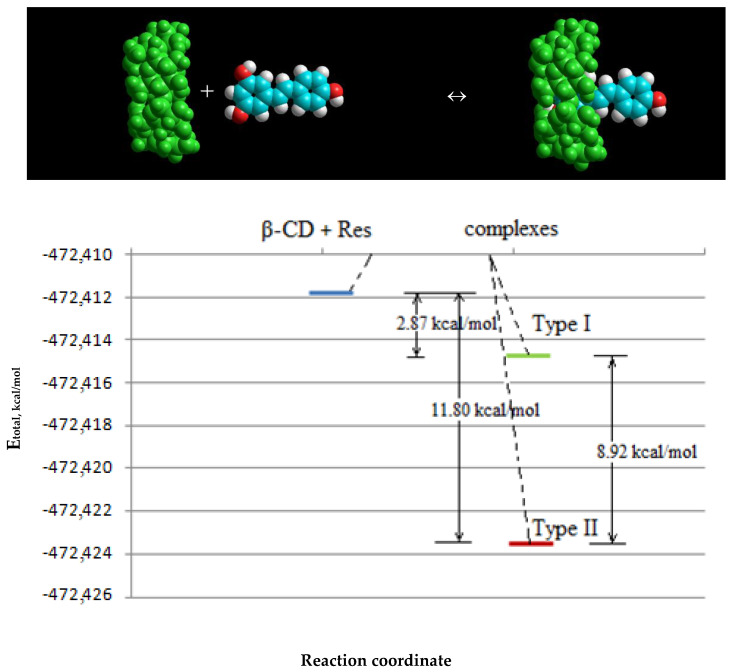
The total amount of energy for the resveratrol–β-CD system before and after complexation.

It can be seen from the data in Table 1 that the complexation energy of the studied complexes ranged from 2.87 to 39.421 kcal/mol. It should be noted that the energy of formation of the 2:1 complex was more than three times higher than for the 1:1 complex. This suggests that the 2:1 complex is more stable and can be formed more easily. As follows from the data presented in the table, the initial state of the system lies higher in energy on the energy curve compared to the resulting inclusion complex (Figure 6).

The data presented in Table 1 and Figure 6 demonstrate that the difference in the energy of individual resveratrol and β-CD molecules and the complexes is about 2.87–11.80 kcal/mol. This excess energy should be released from the system during the formation of the complex, due to the process of complex formation in the β-CD–resveratrol system.

### 2.2. Molecular Dynamics Simulations

**Figure 7:**
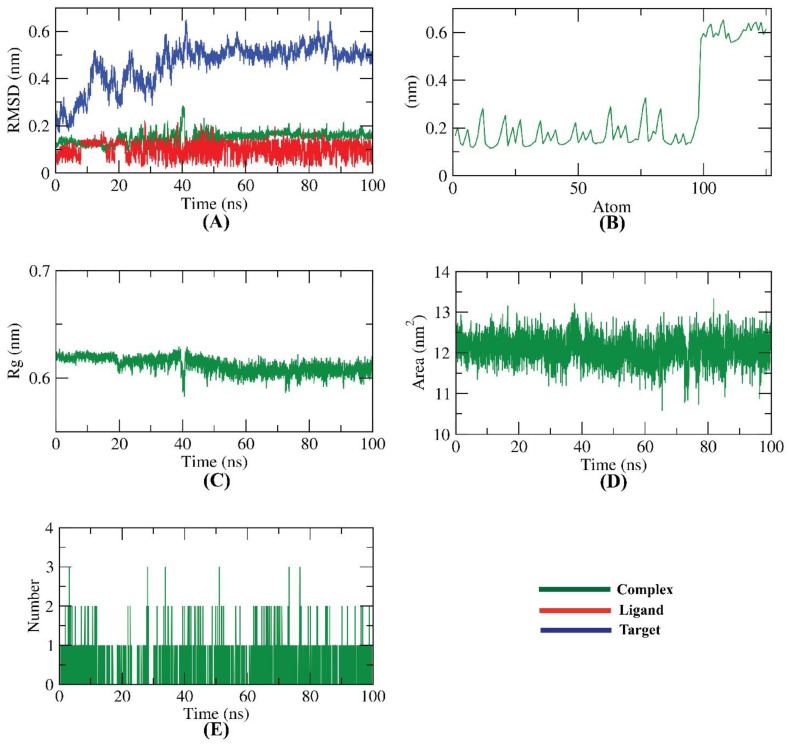
Results of MD simulations of the resveratrol–β-CD complex: (**A**) RMSD, (**B**) RMSF, (**C**) R_g_, (**D**) SASA, and (**E**) H bonding.

The dynamic conformational changes of the resveratrol–β-CD complex were investigated by several MD simulation studies. Initially, RMSD was estimated to expose the stability of the resveratrol–β-CD complex upon both initial and bonding states over a time period of 100 ns. Interestingly, the resveratrol–β-CD complex was stable, exhibiting no major oscillation until the end of the 100 ns (Figure 7A). Secondly, the flexibility of the resveratrol–β-CD complex was studied over an atomic resolution employing the RMSF. The RMSF computation exposed the regions in the target molecule (CD) that fluctuated after binding. Figure 7B illustrates that the binding of resveratrol makes the β-CD slightly flexible in 100–120-atom (CD) areas. Additionally, the compactness of the resveratrol–β-CD was represented by the radius of gyration (R_g_). As Figure 7C indicates, the R_g_ of the resveratrol–β-CD complex was lower at the end of the 100 ns than at the starting time, indicating the stability and compactness of the resveratrol–β-CD system. Furthermore, the interaction between the resveratrol–β-CD complex and the encompassing solvents was examined by solvent-accessible surface area (SASA) over 100 ns. SASA values are an essential indicator of the conformational transformations that eventuate after the binding of components of any complexity. Interestingly, as shown in Figure 7D, the β-CD exhibited a decrease in its surface area, demonstrating a relatively stable SASA value after 100 ns of simulation.

Hydrogen bonds that were included in the resveratrol–β-CD complex were investigated, as they are a very vital factor in the stability of the complex. Figure 7E shows that the highest number of conformations of the β-CD formed up to three hydrogen bonds with resveratrol.

### 2.3. MMPBSA

**Figure 8:**
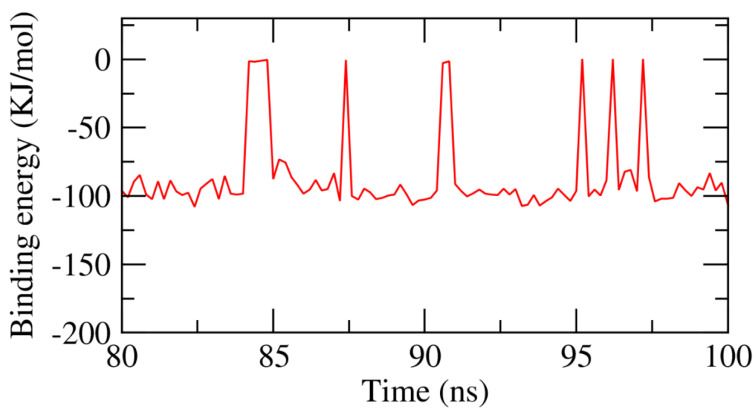
MM-PBSA study of the resveratrol–β-CD complex.

The precise binding free energy of the resveratrol–β-CD complex was studied during the last 20 ns of the MD simulation study, employing a 100 ps interval from MD trajectories, depending on the MM/PBSA method. Furthermore, the MmPbSaStat.py script was employed to estimate the average binding free energy as well as the standard deviation and or the standard error of the outputted files from the g_mmpbsa. As demonstrated in Figure 8, resveratrol displayed binding free energy of −86 KJ/mol with the β-CD.

The obtained data confirmed the binding of the resveratrol–β-CD complex through the optimal kinetics, ideal energy, and correct conformational changes.

### 2.4. Thermographic Measurements

**Figure 9:**
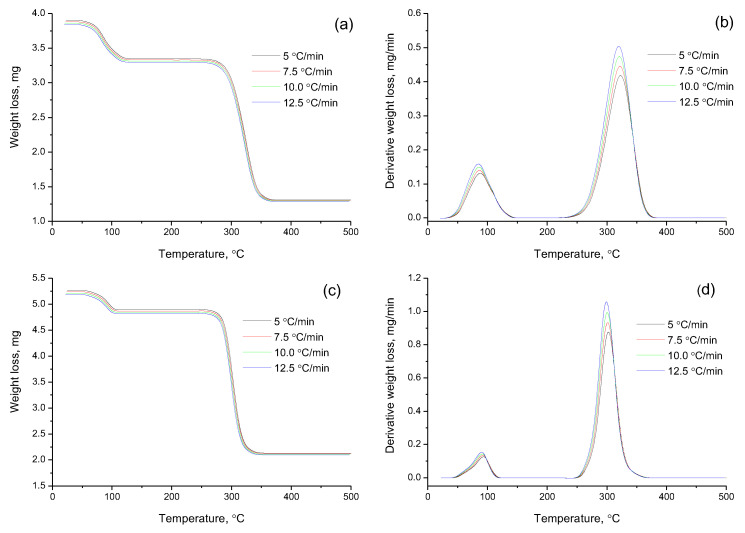
TG curves of the analysis of β-CD–resveratrol inclusion complexes that were obtained at ratios of 2:1 (**a**,**b**) and 4:1 (**c**,**d**) at heating rates from 5 to 12.5 °C/min.

DSC characterization was employed to examine the crystallinity and physical state of resveratrol in the β-CD complex system through the variation of both temperature and heat flow at phase transitions [22]. To estimate the decomposition temperature of β-cyclodextrin inclusion complexes with resveratrol at ratios of 2:1 and 4:1, we performed a thermogravimetric analysis. In order to obtain reliable kinetic analysis results, according to the recommendations of the ICTAC Kinetics Committee [23], the decomposition process was carried out at four different heating rates (Figure 9). The thermoanalytical parameters of the decomposition of β-cyclodextrin with resveratrol at ratios of 2:1 and 4:1 are represented by the TG (mass change) and DTG (rate of mass change) curves.

According to the experimental data of the thermogravimetric analysis, it can be seen that the thermal decomposition of the inclusion complexes of β-cyclodextrin with resveratrol at ratios of 2:1 and 4:1 occurred with slight differences. As follows from the data in Figure 9a, when the β-CD–resveratrol clathrate was heated (2:1), a noticeable decrease in mass began at a temperature of ~50 °C. This temperature value refers to the release of water molecules from the inner cavity of the clathrate [24]. In the case of β-CD–resveratrol (4:1) (Figure 9a,b), the endothermic peak (T_onset_ = 50 °C) associated with water loss was present, but it was less pronounced. Thermograms (Figure 9a,b) of β-CD–resveratrol clathrates show characteristic thermal effects occurring at a temperature of 230–380 °C for β-CD–resveratrol complexes (2:1), and at 250–370 °C for β-CD–resveratrol complexes (4:1).

A comparative analysis of the data shows that the peak heat absorption caused by the activation of thermal degradation of β-cyclodextrin was in the range of 290–380 °C for pure β-cyclodextrin, and 230–380 °C for β-CD–resveratrol clathrates (Figure 9). These data indicate a decrease in the thermal stability of cyclodextrin when resveratrol is included in its cavity. This phenomenon can be considered proof of the formation of stable β-CD–resveratrol inclusion complexes (2:1, 4:1) and strong intermolecular bonds due to the formation of van der Waals hydrophobic forces that break down with increasing temperatures, while the β-CD molecule itself begins to break down at a higher temperatures (T_onset_ = 290 °C) (Figure 9). Based on this information, the thermal decomposition reactions and recrystallization changes were as follows:

β-CD–resveratrol **·**H_2_O (s) → β-CD–resveratrol (s) + H_2_O (g);

β-CD–resveratrol (s) → β-CD (s) + resveratrol (s);

β-CD (s) → CO_2_ (g) + H_2_O (g).

**Figure 10:**
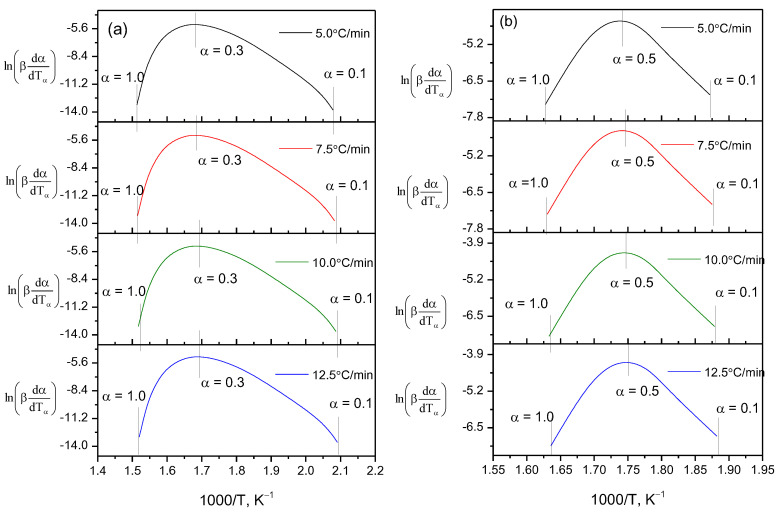
Logarithmic dependence $\ln\left[\beta \frac{d\alpha }{dT_{\alpha }}\right]$ on 1/T, constructed according to thermogravimetric analysis: (**a**) β-CD–resveratrol (2:1) and (**b**) β-CD–resveratrol (4:1) (Friedman method) at heating rates from 5 to 12.5 °C/min.

**Figure 11:**
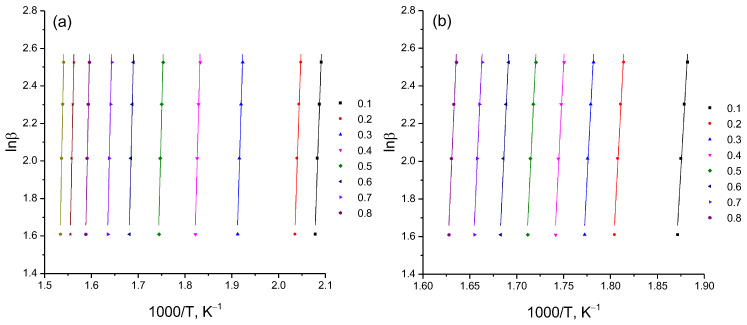
Logarithmic dependence of lnβ on 1/T, constructed according to thermogravimetric analysis (**a**) β-CD–resveratrol (2:1) and (**b**) β-CD–resveratrol (4:1) (Flynn–Ozawa–Wall method) at heating rates from 5 to 12.5 °C/min.

An objective analysis of the process of thermal destruction of β-CD–resveratrol inclusion complexes (2:1, 4:1) is possible when comparing the activation energies of the process, since the activation energy is a reliable criterion by which a direct comparison can be made. From a large number of conversion methods, we selected the Ozawa–Flynn–Wall (OFW) [24] and Friedman (FR) methods [25]. The main difference between these methods and others is that in order to calculate the kinetic parameters, it is not necessary to know the order of the thermal destruction process beforehand. Calculated data on the thermal destruction of β-CD–resveratrol clathrates (2:1, 4:1) are presented in Table 2, on the basis of which logarithmic dependences and lnβ on 1/T were constructed by the OFU method [26] (Figure 10), and lnβdαdTα from 1/T according to the Friedman method [24] (Figure 11).

**Figure 12:**
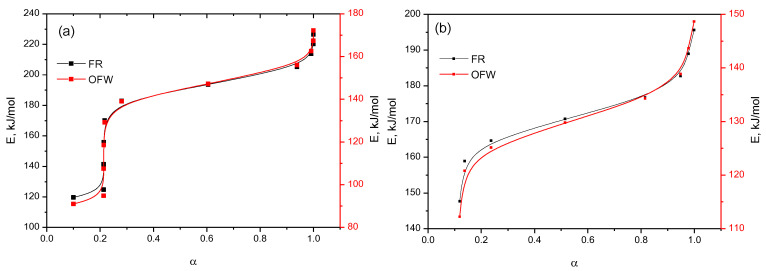
Dependences of activation energy (Ea) on the degree of transformation (α) for (**a**) β-CD–resveratrol (2:1) and (**b**) β-CD–resveratrol (4:1).

As can be seen from Figure 10 and Figure 11, using various approximations, a wide range of bioconversion methods can be obtained. The calculation results that we obtained (Table 2 and Figure 12) clearly show the effectiveness of these methods.

The analysis of the dependence of E*_α_* on *α* shows (Figure 12a) that with an increase in the degree of transformation, the activation energy value increases from 110 to 130 kJ/mol in the OFU method, and from 120 to 170 kJ/mol in the FR method. Such a character of the activation energy values indicates a change in the reaction mechanism as they proceed, which can be explained by at least two reasons. At the beginning of the process, the value of E*_α_* = 110 kJ/mol at *α* = 0.1, which indicates the thermal decomposition of several atomic layers adjacent to the surface of the solid. With an increase in the degree of transformation from 0.1 to 0.9, the reaction rate increases rapidly, reaching a maximum value at the inflection point. Similarly, the thermal degradation of β-CD–resveratrol inclusion complexes (4:1) occurs (Figure 12b).

For kinetic analysis of the process of thermal destruction of the studied sample, a nonparametric kinetic (NPK) method was applied as well [27]. Response time experimental values were found in the matrix, which were expressed as two vectors’ multiplication, containing the information on k(T) and f(α). This mathematical model is a consequence of Equation (1):

(1) $$r=f\left(\alpha \right)\cdot k\left(T\right)$$

The NPK method applies the algorithm of singular value decomposition (SVD) for the matrix *M*’s decomposition into two vectors [27]. Matrix *M* is analyzed as follows:

(2) $$M=U\left(diag\cdot S\right)\cdot V^{T}$$

**Figure 13:**
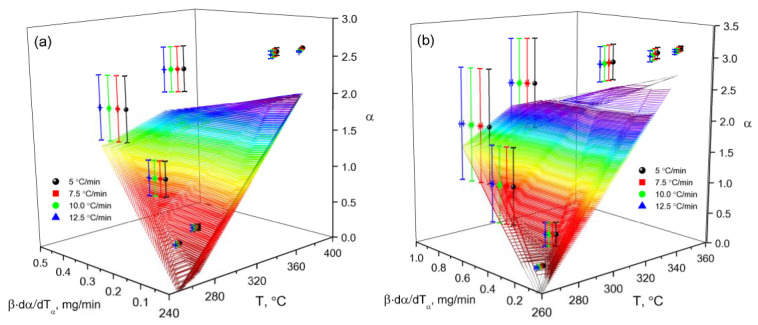
Surface in three-dimensional space: the dependence of the reaction rate (β·d*α*/dT_*α*_) on the temperature (T) and the degree of transformation (*α*) in the air—(**а**) β-CD–resveratrol (2:1) and (**b**) β-CD–resveratrol (4:1).

The reaction model $g\left(\alpha \right)$ is dependent on the conversion degree, while $f\left(T\right)$ is dependent on temperature. Reaction velocity $\beta d\alpha /{dT}_{\alpha }$ was explored by different experiments at several heating rates, β, and was interpolated as a surface in 3D space (β*d*α/*dT_α_*, *α*, *Т*) (Figure 13). This surface was organized as a matrix, where the lines represent the conversion degrees from *α*_1_ to *α*_j_, and columns represent temperatures from *T*_i_ to *T*_j_. The results of the kinetic analysis are shown in Figure 13 and Table 3.

According to the obtained data of thermogravimetric analysis, all three methods were satisfactory for calculation $\bar{E}$ within the required accuracy.

### 2.5. Morphology of β-CD Particles and the Binary System β-CD–Resveratrol

**Figure 14:**
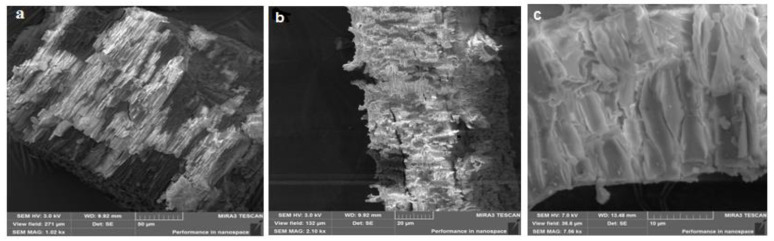

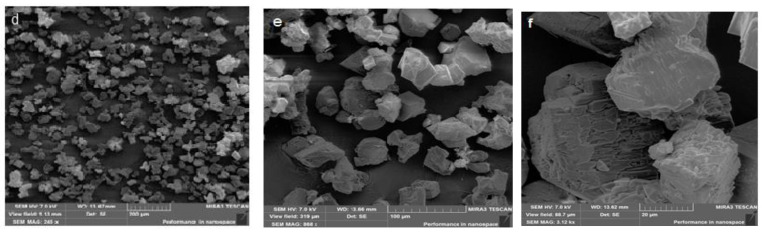
Scanning electron micrographs of β-CD (**a**–**c**) and the β-CD–resveratrol (2:1) inclusion complex (**d**–**f**) at various magnifications.

SEM is an important tool for measuring surface roughness and visualizing the surface texture of components [28]. The morphology of both β-CD particles and the β-CD–resveratrol binary system was examined using SEM. Figure 14 displays scanning electron micrographs of the β-CD–resveratrol inclusion complex (2:1). The studied clathrate samples were previously sprayed with a conductive carbon layer. The images were procured at accelerating voltages of 3 and 7 kV. The magnification in Figure 14a–c is from 1010 to 7560×, while that in Figure 14d–e is from 931 to 7000×.

Scanning electron micrographs showed the formation of crystals for inclusion complexes. The obtained images (Figure 14a–c) showed the layered structure of β-cyclodextrin, while the samples of β-CD–resveratrol (2:1) (d–f) showed a sharp change in the shape and morphology of the imaged crystals. The crystallization of raw materials was observed, the layered structure was not visible, and the shape of the crystals was different and was covered with a film.

### 2.6. FTIR and ^1^H NMR Measurements

**Figure 15:**
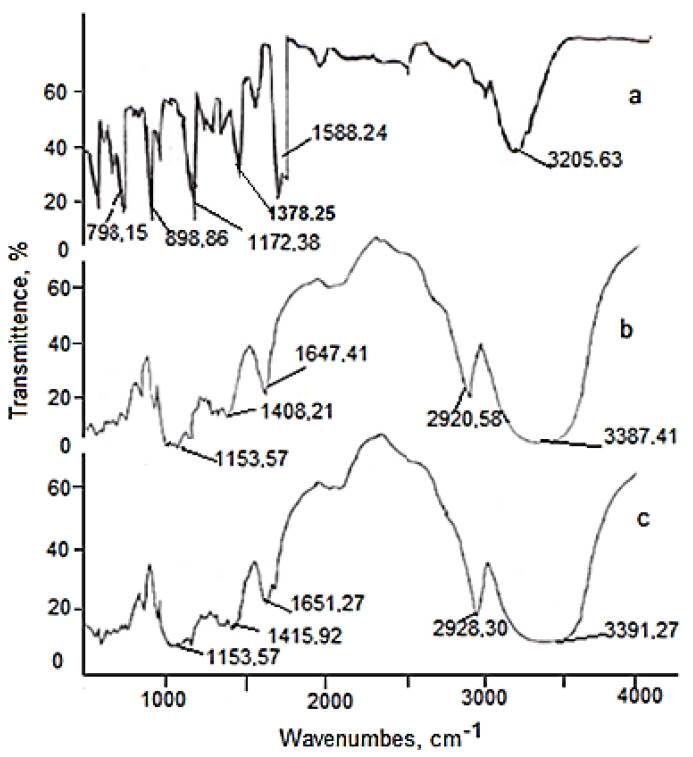
FTIR spectra of resveratrol (**а**), β-CD (**b**) and β-CD–resveratrol (2:1) (**с**).

The combined use of various methods of describing the objects under study, depending on the physical condition under consideration, currently gives the best results in terms of model reliability. The infrared spectra of resveratrol, β-CD, and the inclusion complex β-CD–resveratrol (2:1) are shown in Figure 15. In the FTIR spectra of β-CD and β-CD–resveratrol, valence bond oscillations of the hydroxyl group O-H had the form of a wide band, with maxima at 3387 cm^−1^ and 3391 cm^−1^, respectively. Infrared absorption of resveratrol in the range of 1700–450 cm^−1^ showed three characteristic intense bands at 1610, 1588, and 1378 cm^−1^, corresponding to the absorption of the aromatic C-C double bond, the absorption of the olefin C-C bond, and the absorption of C-O, respectively. The FTIR spectra of inclusion compounds showed changes in the spectral characteristics of the “guest” molecule—in fact, the intensity of the bands at 1378 cm^−1^ and 970 cm^−1^ decreased, while the bands at 1610 cm^−1^ and 1588 cm^−1^ disappeared. These facts may be related to the formation of inclusion complexes [28,29].

The ^1^H and ^13^C NMR spectroscopic methods are among the most informative methods for confirming the formation of inclusion complexes [30]. In the β-CD structure, protons H3 and H5 are located inside a conical cavity—in particular, H3 is located next to a wider edge, while H5 is located next to a narrower edge, while the remaining protons H1, H2, and H4 are located on the outer surface of the β-CD molecule [29]. This method allows registering a pronounced chemical shift of protons H3 and H5 in β-CD (Δδ = δ − δ_0_). They are oriented inside the cavity of the torus, which is due to the placement of the “guest” molecule in the hydrophobic cavity of cyclodextrin. In the ^1^H-NMR spectrum of β-CD–resveratrol (2:1), the greatest difference in the values of the chemical shift (Δδ, ppm) was characteristic of the intraspheric protons H3 (Δδ = −0.094) and H5 of β-CD (Δδ = −0.015) (Table 4). This allowed us to conclude about the formation of an internal (inclusive) complex in clathrate. A proportional increase in the chemical shift in the ^1^H-NMR vibrational spectra was observed with an increase in the concentration of “guest” compounds (resveratrol) due to a shift in the equilibrium state towards the formation of an inclusion complex. These conclusions are consistent with the data of the authors [30,31].

### 2.7. Antioxidant Properties of Resveratrol and β-CD–Resveratrol (2:1)

Table 5 shows the antioxidant properties of resveratrol and β-CD–resveratrol. It was noted that resveratrol, as along with its inclusion complex (IC), has very strong antioxidant activity, which is consistent with the data previously described in [32]. The ability to eliminate the DPPH radical in the inclusion complex was somewhat reduced after resveratrol was encapsulated by β-CD. A slight decrease in the IC_50_ in β-CD–resveratrol (2:1) indicates the enhancement in the antioxidant activity. All reported data were repeated three times (Table 5). Thus, the formed IC retained the antioxidant activity of resveratrol, meaning that the complexation increased the antioxidant activity and, accordingly, can potentially be used in the food industry to improve the shelf life of products.

## 3. Material and Methods

The utilized materials as well as the detailed methods of (Molecular modeling, Docking simulation, Molecular dynamics simulations, Preparation of resveratrol inclusion complexes with β-cyclodextrin, Thermal properties, Spectroscopic measurements of inclusion complexes, Antioxidant activity of Resveratrol and β-CD:Resveratrol) are presented in the Supplementary Materials.

## 4. Conclusions

This paper presents the results of a study of the encapsulation of resveratrol with β-cyclodextrin. The use of microwave treatment in an aqueous–alcohol medium made it possible to obtain resveratrol inclusion complexes with β-cyclodextrin at high yields. The molecular docking experiments indicated the good binding between resveratrol and β-cyclodextrin. Then, five MD simulations and MM-PBSA experiments confirmed the accuracy of that binding. The results of thermographic measurements with a differential scanning calorimeter were obtained. Using the Ozawa–Flynn–Wall and Friedman methods, as well as the method of nonparametric kinetics, the values of the activation energy of thermo-oxidative destruction of resveratrol clathrate complexes were calculated. The kinetic parameters of thermal decomposition obtained by various methods were in satisfactory agreement, and confirmed the reliability of the results. Molecular modeling of resveratrol inclusion complexes with β-cyclodextrin was carried out. The total energy of the studied systems was estimated by the semi-empirical method AM1. It was shown that complexes with a ratio of 1:2 are more stable and can be formed more easily than complexes with a ratio of 1:1. The structure and physicochemical properties of the inclusion complexes were systematically determined by ^1^H and ^13^C NMR, FTIR spectra, DSC, and SEM. Both resveratrol and its inclusion complexes showed good antioxidant activity. It was shown that encapsulation with β-cyclodextrin leads to the enhancement of the antioxidant activity (IC_50_) of resveratrol in the clathrate complex (2:1).

## Figures and Tables

**Table 1:** Calculated values of the complexation energy of the studied inclusion complexes of β-CD: resveratrol.

Complex	E_total_, kcal/mol		ΔЕ_complex_, kcal/mol
	Initial Substances	Complex	
Composition 1:1 Type I	−472,411.8326	−472,423.6333	11.80066
Composition 1:1 Type II	−472,411.8326	−472,414.7083	2.8757
Composition 2:1	−877,529.7433	−877,569.1649	39.42156

**Table 2:** Kinetic parameters of the thermal degradation of β-CD–resveratrol clathrates (2:1, 4:1).

Sample	${\bar{E}}_{OFW}$, kJ/mol	$\ln{\bar{A}}_{OFW},\;c^{-1}$	${\bar{E}}_{FR},\;\mathrm{kJ}/\mathrm{mol}$	$\ln{\bar{A}}_{FR},\;c^{-1}$
β-CD–resveratrol (2:1)	135.03	2.06 × 10^3^	177.65	7.12 × 10^20^
β-CD–resveratrol (4:1)	131.68	1.08 × 10^3^	173.25	1.65 × 10^17^

**Table 3:** Kinetic parameters of the thermal decomposition of β-CD–resveratrol (2:1, 4:1), calculated by the method of nonparametric kinetics (NPK).

Sample	λ	$\bar{E},\;\mathrm{kJ}/\mathrm{mol}$	$\mathrm{lnA},\;{с}^{-1}$
β-CD–resveratrol (2:1)	5.4	177.43	6.85 × 10^16^
β-CD–resveratrol (4:1)	5.0	173.24	3.15 × 10^15^

**Table 4:** ^1^Н and ^13^С NMR chemical shift values for β-CD in the absence and the presence of β-CD (molar ratio 2:1).

Proton	δ_0_, ppm (β-CD)		δ, ppm (β-CD: Resveratrol)		Δδ (δ − δ_0_), ppm	
	δ (^1^Н)	δ (^13^С)	δ (^1^Н)	δ (^13^С)	δ (^1^Н)	δ (^13^С)
H-1	4.780	102.416	4.770	102.493	−0.010	0.076
H-2	3.312	72.853	3.268	72.974	−0.044	0.121
H-3	3.452	73.514	3.358	73.608	−0.094	0.094
H-4	3.283	82.021	3.267	82.125	−0.016	0.104
H-5	3.330	72.493	3.315	72.595	−0.015	0.102
H-6	3.58	60.408	3.57	60.492	−0.017	0.084

**Table 5:** Antioxidant activity of resveratrol and β-CD–resveratrol ^a^.

Samples	IC_50_ (μg/mL)
Resveratrol	14.3 ± 0.4
β-CD–resveratrol (2:1)	12.1 ± 2.3

^a^ All of the data obtained were repeated three times (*p* ≤ 0.05).

## Data Availability

Data are available with the corresponding authors upon request.

## Supplementary Materials

The following supporting information can be downloaded at: https://www.mdpi.com/article/10.3390/plants11131678/s1, ^1^H and ^13^C -NMR spectra of free (a) β-CD and (b) β-CD:Res (2:1) inclusion complex besides the details of method.

## References

[1] Frémont L. (2000). Biological effects of resveratrol. Life Sci..

[2] Samuel V.P., Gupta G., Dahiya R., Jain D.A., Mishra A., Dua K. (2019). Current update on preclinical and clinical studies of resveratrol, a naturally occurring phenolic compound. Critical Reviews™ in Eukaryotic Gene Expression.

[3] Wang B., Bellot G.L., Iskandar K., Chong T.W., Goh F.Y., Tai J.J., Schwarz H., Wong S.C., Pervaiz S. (2020). Resveratrol attenuates TLR-4 mediated inflammation and elicits therapeutic potential in models of sepsis. Sci. Rep..

[4] Giordo R., Zinellu A., Eid A.H., Pintus G. (2021). Therapeutic potential of resveratrol in COVID-19-associated hemostatic disorders. Molecules.

[5] Li L., Qiu R.L., Lin Y., Cai Y., Bian Y., Fan Y., Gao X.J. (2018). Resveratrol suppresses human cervical carcinoma cell proliferation and elevates apoptosis via the mitochondrial and p53 signaling pathways. Oncol. Lett..

[6] Tong W., Chen X., Song X., Chen Y., Jia R., Zou Y., Li L., Yin L., He C., Liang X. (2020). Resveratrol inhibits LPS-induced inflammation through suppressing the signaling cascades of TLR4-NF-κB/MAPKs/IRF3. Exp. Ther. Med..

[7] Ferraz da Costa D.C., Fialho E., Silva J.L. (2017). Cancer chemoprevention by resveratrol: The p53 tumor suppressor protein as a promising molecular target. Molecules.

[8] Ren B., Kwah M.X.-Y., Liu C., Ma Z., Shanmugam M.K., Ding L., Xiang X., Ho P.C.-L., Wang L., Ong P.S. (2021). Resveratrol for cancer therapy: Challenges and future perspectives. Cancer Lett..

[9] Patra S., Pradhan B., Nayak R., Behera C., Rout L., Jena M., Efferth T., Bhutia S.K. (2021). Chemotherapeutic efficacy of curcumin and resveratrol against cancer: Chemoprevention, chemoprotection, drug synergism and clinical pharmacokinetics. Semin. Cancer Biol..

[10] Valentovic M.A. (2018). Evaluation of resveratrol in cancer patients and experimental models. Adv. Cancer Res..

[11] Santos A.C., Pereira I., Magalhães M., Pereira-Silva M., Caldas M., Ferreira L., Figueiras A., Ribeiro A.J., Veiga F. (2019). Targeting cancer via resveratrol-loaded nanoparticles administration: Focusing on in vivo evidence. AAPS J..

[12] Szulc-Musioł B., Sarecka-Hujar B. (2021). The use of micro-and nanocarriers for resveratrol delivery into and across the skin in different skin diseases—A literature review. Pharmaceutics.

[13] Lin M.-H., Hung C.-F., Sung H.-C., Yang S.-C., Yu H.-P., Fang J.-Y. (2021). The bioactivities of resveratrol and its naturally occurring derivatives on skin. J. Food Drug Anal..

[14] Vestergaard M., Ingmer H. (2019). Antibacterial and antifungal properties of resveratrol. Int. J. Antimicrob. Agents.

[15] Annunziata G., Maisto M., Schisano C., Ciampaglia R., Narciso V., Tenore G.C., Novellino E. (2018). Resveratrol as a novel anti-herpes simplex virus nutraceutical agent: An overview. Viruses.

[16] Cottart C.H., Nivet-Antoine V., Laguillier-Morizot C., Beaudeux J.L. (2010). Resveratrol bioavailability and toxicity in humans. Mol. Nutr. Food Res..

[17] Ma D.S., Tan L.T.-H., Chan K.-G., Yap W.H., Pusparajah P., Chuah L.-H., Ming L.C., Khan T.M., Lee L.-H., Goh B.-H. (2018). Resveratrol—potential antibacterial agent against foodborne pathogens. Front. Pharmacol..

[18] Hashemi M., Hashemi M., Amiri E., Hassanzadazar H., Daneshamooz S., Aminzare M. (2019). Evaluation of the synergistic antioxidant effect of resveratrol and Zataria multiflora boiss essential oil in sodium alginate bioactive films. Curr. Pharm. Biotechnol..

[19] Schlich M., Lai F., Pireddu R., Pini E., Ailuno G., Fadda A., Valenti D., Sinico C. (2020). Resveratrol proniosomes as a convenient nanoingredient for functional food. Food Chem..

[20] Santos A.C., Veiga F., Ribeiro A.J. (2011). New delivery systems to improve the bioavailability of resveratrol. Expert Opin. Drug Deliv..

[21] Sapino S., Carlotti M.E., Caron G., Ugazio E., Cavalli R. (2009). In silico design, photostability and biological properties of the complex resveratrol/hydroxypropyl-β-cyclodextrin. J. Incl. Phenom. Macrocycl. Chem..

[22] Zou A., Zhao X., Handge U.A., Garamus V.M., Willumeit-Römer R., Yin P. (2017). Folate receptor targeted bufalin/β-cyclodextrin supramolecular inclusion complex for enhanced solubility and anti-tumor efficiency of bufalin. Mater. Sci. Eng. C.

[23] Gallagher P.K., Brown M.E. (2003). Handbook of Thermal Analysis and Calorimetry.

[24] Pereira A.N., Trevisan O.V. (2014). Thermoanalysis and reaction kinetics of heavy oil combustion. J. Braz. Soc. Mech. Sci. Eng..

[25] Freeman E.S., Carroll B. (1958). The application of thermoanalytical techniques to reaction kinetics: The thermogravimetric evaluation of the kinetics of the decomposition of calcium oxalate monohydrate. J. Phys. Chem..

[26] Flynn J.H., Wall L.A. (1966). A quick, direct method for the determination of activation energy from thermogravimetric data. J. Polym. Sci. Part B Polym. Lett..

[27] Serra R., Nomen R., Sempere J. (1998). The non-parametric kinetics a new method for the kinetic study of thermoanalytical data. J. Therm. Anal. Calorim..

[28] Maazaoui R., Abderrahim R. (2015). Applications of cyclodextrins: Formation of inclusion complexes and their characterization. Int. J. Adv. Res..

[29] Zhao D., Liao K., Ma X., Yan X. (2002). Study of the supramolecular inclusion of β-cyclodextrin with andrographolide. J. Incl. Phenom. Macrocycl. Chem..

[30] Chen W., Yeo S.C.M., Elhennawy M.G.A.A., Lin H.-S. (2016). Oxyresveratrol: A bioavailable dietary polyphenol. J. Funct. Foods.

[31] Zhang J.-Q., Wu D., Jiang K.-M., Zhang D., Zheng X., Wan C.-P., Zhu H.-Y., Xie X.-G., Jin Y., Lin J. (2015). Preparation, spectroscopy and molecular modelling studies of the inclusion complex of cordycepin with cyclodextrins. Carbohydr. Res..

[32] Lu Z., Chen R., Liu H., Hu Y., Cheng B., Zou G. (2009). Study of the complexation of resveratrol with cyclodextrins by spectroscopy and molecular modeling. J. Incl. Phenom. Macrocycl. Chem..

