# Supplementary material for: Combined In Silico and Experimental Investigations of Resveratrol Encapsulation by Beta-Cyclodextrin
Source: Plants (Basel). 2022 Jun 24;11(13):1678. doi: 10.3390/plants11131678 (PMC9269368; doi:10.3390/plants11131678)
Supplement: Supplementary file 1 [file plants-11-01678-s001.zip › plants-1773341-supplementary.pdf]

**Supporting data**

# Combined *In silico* and experimental investigations of resveratrol encapsulation by beta-cyclodextrin

**Ainara Iskineyeva[1], Serik Fazylov[2], Ryszhan Bakirova[3], Akmaral Sarsenbekova[2], Irina Pustolaikina[2], Olzhas Seilkhanov[4], Aisha A. Alsfouk[5], Eslam B. Elkaeed[6], Ibrahim H. Eissa[7], Ahmed M. Metwaly *[8,9]**

[1]Saken Seifullin Kazakh Agrotechnical University, 010000, Nur-Sultan, Kazakhstan

[2]Karagandy University of the name of academician E.A. Buketov, Kazakhstan, 100024, Kazakhstan

[3]Karaganda Medical University, 100012, Karaganda, Kazakhstan

[4]Kokshetau State University, 020000, Kokshetau, Kazakhstan

[5] Department of Pharmaceutical Sciences, College of Pharmacy, Princess Nourah bint Abdulrahman University, P.O. Box 84428, Riyadh 11671, Saudi Arabia aaalsfouk@pnu.edu.sa
[6]Department of Pharmaceutical Sciences, College of Pharmacy, AlMaarefa University, Riyadh 13713, Saudi Arabia ikaeed@mcst.edu.sa
[7] Pharmaceutical Medicinal Chemistry & Drug Design Department, Faculty of Pharmacy (Boys), Al-Azhar University, Cairo11884, Egypt ibrahimeissa@azhar.edu.eg
[8] Pharmacognosy and Medicinal Plants Department, Faculty of Pharmacy (Boys), Al-Azhar University, Cairo, Egypt ametwaly@azhar.edu.eg
[9]Biopharmaceutical Product Research Department, Genetic Engineering and Biotechnology Research Institute, City of Scientific Research and Technological Applications, Alexandria, Egypt

**Content**


| | |
|---|---|
| Method | Materials |
| | Molecular modeling |
| | Docking simulation |
| | Molecular dynamics simulations |
| | Preparation of resveratrol inclusion complexes with β-cyclodextrin |
| | Thermal properties |
| | Spectroscopic measurements of inclusion complexes |
| | Antioxidant activity of Resveratrol and β-CD:Resveratrol |
| Spectra | $^{1}$H-NMR spectra of free (a) β-CD and (b) β-CD:Res (2:1) inclusion complex |
| | $^{13}$C-NMR spectra of free (a) β-CD and (b) β-CD:Res (2:1) inclusion complex |

# Method

## 1. Materials.

The following reagents were used in the experiments: β-cyclodextrin (99.0%, purchased from Sigma–Aldrich), white crystalline substance, melting point 283-315°C; resveratrol (98%, China), white crystalline substance, $C_{14}H_{12}O_3$, mol. mass 228.25 g/mol, melting point 253-255°C, solubility in water 3 mg/100ml. β-Cyclodextrin is well soluble in organic solvents: ethanol, dimethylformamide and dimethyl sulfoxide (65 mg/ml). 1,1-Diphenyl-2-picrylhydrazyl (DPPH) were purchased from Sigma–Aldrich

## 2. Molecular modeling

### 2.1. Preparation of the β CD structure

The 3D crystal structures of the β CD were extracted from the cyclo/maltodextrin/ alpha-cyclodextrin complex, beta-cyclodextrin complex, and gamma cyclodextrin complex, respectively. These complexes were retrieved from RCSB Protein Data Bank with PDB IDs of 1BFN (resolution 2.07 Å). The docking analyses were performed using Discovery Studio 4.0 software to evaluate the free energies and binding mode of Roseofungin molecule with the core site of β CD. The most promising pose were selected depending the increased binding free energy (ΔG).

The 3D crystal structures of β CD were prepared by selecting β CD subunits and removing protein and all water molecules, heteroatoms and co-factors. Moreover, the correction of uncorrected valence atoms and crystallographic disorders were performed using alternate conformations and valence monitor options. Then, the β CD were protonated and its inflexibility was obtained by creating fixed atom constraint. Next, the energy was energy minimized by applying CHARMM (Chemistry at HARvard Macromolecular Mechanics) force fields, and MMFF94 (Merck Molecular force field) force field for charge and partial charge, respectively. The binding sites of the β CD were defined as receptor molecules and prepared for docking.

### 2.2. Preparation of ligand

The 2D structure of anabazine alkaloid molecule was sketched using ChemBioDraw Ultra 14.0 and saved in MDL-SD file format. Then, the SD file was opened (by Discovery studio 4.0 software) and protonated. Force fields were applied on the molecule to get lowest energy minimum structure via CHARMM and MMFF94 force fields for charge and partial charge, respectively. Then, each of them was prepared for docking by applying ligand preparation protocol.

## 2.3. Docking simulation

The docking is a technique that can reliably predict the preferred configuration of one molecule relative to another molecule when they are bound to each other to forma stable complex. The evaluation of generated poses was mainly based on the number of interactions they formed with the residues of active site upon binding.

The molecular docking of anabazine alkaloid was performed using CDOCKER protocol which is an implementation of the CDOCKER algorithm. CDOCKER is a grid-based molecular docking method that employs CHARMM-based molecular dynamics (MD) scheme to dock ligands into a receptor binding site . The default values were selected for the CDOCKER protocol. The CDOCKER energy (receptor-ligand interaction energy) of best docked poses was calculated.

## 2.4. Molecular modeling

Molecular modeling of resveratrol inclusion complexes with β-CD was carried out by the semi-empirical AM1 method using the HyperChem 8.0 program. The chemical structures were taken from the PubChem Substances and Compounds database (pubchem.ncbi.nlm.nih.gov): CID 444041 for beta-cyclodextrin, CID 445154 for trans-resveratrol, CID 164233321 for cis-resveratrol [42]. Initially, the structure of the complex was constructed, then its geometry was optimized by the AM1 method, geometric and energy parameters were evaluated. All calculations were carried out for gas phase without taking into account the influence of the solvent.

## 2.5. Molecular dynamics simulations

Molecular dynamics simulation of the protein-ligand complexes was performed using GROMACS 2021.1 version and Linux 5.4 package. The GROMOS96 54a7 forcefield was selected as the force field for proteins and the ligand topologies were generated from the PRODRG[2] server. All the complexes were solvated using simple point charge (SPC) water molecules in a rectangular box. To make the simulation system electrically neutral, required number of Na+ and Cl− ions were added while 0.15 mol/L salt concentrations were set in all the systems. Using the steepest descent method, all the solvated systems were subjected to energy minimization for 5000 steps. Afterwards, NVT (constant number of particles, volume, and temperature) series, NPT (constant number of particles, pressure, and temperature) series, and the production run were conducted in the MD simulation. The NVT and the NPT series were conducted at a 300 K temperature and 1

atm pressure for the duration of 300 ps. V-rescale thermostat and Parrinello-Rahman barostat were selected of the performed simulation. Finally, the production run was performed at 300 K for a duration of 100 ns (nanoseconds). Thereafter, a comparative analysis was performed measuring root mean square deviation (RMSD), root mean square fluctuation (RMSF), radius of gyration (Rg), solvent accessible surface area (SASA) and hydrogen bonds to analyze their stability. The Xmgrace program was used to represent the analyses in the form of plots.

**3. Preparation of resveratrol inclusion complexes with β-cyclodextrin**

Inclusion complexes (IC) β-CD: resveratrol (2:1 and 4:1) were prepared in a water-alcohol medium (1:1) in microwave activation conditions [41]. β-CD (0.04 mmol) was dissolved in distilled water at temperature of 60°C in a water bath until completely dissolved. The solution was cooled to 50°C and, with stirring, an alcoholic solution of vitamin Resveratrol (0.02 mmol) was added drop wise. Then the mixture was subjected to microwave irradiation for 4 minutes (8x0.5 minutes in 2-minute increments) at 80°C using the «Anton Paar Monowave 300» device. The temperature of the reaction medium was controlled by an IR-sensor method. After the end of the reaction, a gradual turbidity of the solution and the formation of small particles of the β-CD: resveratrol inclusion complex were observed. The resulting solution was cooled in a refrigerator to 5°C and centrifuged to separate the clathrates. The precipitate was then centrifuged and dried in vacuum at 60$^{o}$C. Under these conditions, the yields of the inclusion complexes were 82-83%. The resulting dry powder was ground to a homogeneous state in a mortar and stored in hermetically sealed vials in a desiccator with $CaCl_2$. The obtained product was a white powders that dissolves in water to form milky white colloidal solutions.

The yield of the inclusion complex is calculated as the following equation:

$$\text{Yield (\%)} = (W_{IC} / W_{res} + W_{CD}) \times 100$$

where the $W_{IC}$ is the weight of the inclusion complex, $W_{res}$ and $W_{CD}$ is the total weight of Resveratrol and β-CD added into the inclusion complex system, respectively.

## 4. Thermal properties

Samples of β-CD and the inclusion complex with vitamin Resveratrol (weight of the samples 12 mg) were analyzed by thermographic method. The thermal analysis was carried out by the differential thermogravimetry (DTG) and differential scanning calorimetry (DSC) methods using the DTA/DSC differential scanning calorimeter device of the Setaram company. The measurements were carried out in a dynamic mode in the temperature range of 30-500$^oC$ and the heating rate was from 5 to 12,5$^oC$/min in an inert medium( nitrogen), an $Al_2O_3$ crucible. All calculations were performed using the Mathcad program.

## 5. Spectroscopic measurements of inclusion complexes

FT-IR spectra were taken with the Cary 600 Series IR-Fourier spectrometer made by Agilent Technologies (USA) in the range of 4000-400 $cm^{-1}$. The samples were prepared from the tested substances and KBr with the mass ratio of 1:100. The $^1H$, $^{13}C$ NMR measurements were carried out in DMSO-$d_6$ (Aldrich) solutions, other chemicals were of analytical reagent grade purity. NMR spectra of inclusion complexes, cyclodextrin and resveratrol were taken by spectrometer JNM-ECA Jeol 400 operating at 400 MHz (frequency 399.78) using TMS as internal standard. Chemical shifts were expressed as ppm (δ). All measurements were carried out at a resolution of 4.0 $cm^{-1}$, and the number of scans was 40. The melting temperatures of the complexes are measured on the device "Boetius" (Germany).

## 6 Antioxidant activity of Resveratrol and β-CD:Resveratrol

The ability of Resveratrol to scavenge DPPH was assessed according to methods previously reported in the literature [62-64]. Statistical data processing was carried out using Microsoft Excel (2013) according to the formula:

$$\text{Antioxidant activity} = [(A_{DPPH} - A_{Sample})/A_{DPPH}]\text{x}100\%$$

where $A_{DPPH}$ and $A_{Sample}$ are the absorbances of the DPPH solutions in the absence and presence of Resveratrol or Resveratrol:β-CD (1:2) complex, respectively.

To assess the antiradical activity of the studied samples a methanol solution of DPPH (100 µM) was used. For the selection of substances, 2 ml of 100 µM of a methanol solution of DPPH was mixed with 20 µl of the test object dissolved in methanol at a concentration of 5 µM. Thus,

the final concentration of the test substance in the reaction mixture was 50 μM. After mixing the solution of the test compound with DPPH, the samples were transferred to the dark for 30 minutes and the change in optical density was measured at 515 nm. After that, the concentration of the test substance was determined, capable of reducing the optical density of $IC_{50}$ (DPPH) by 50%. All the data obtained were repeated three times ($p \leq 0.05$).

$^{1}H$ and $^{13}$ C-NMR spectra of free (a) β-CD and (b) β-CD:Res (2:1) inclusion complex

The 1H and 13C NMR spectra were taken on a JNM-ECA Jeol400 spectrometer (frequency 399.78 and 100.53 MHz, respectively) using a DMSO-d6 solvent. Chemical shifts were measured relative to the signals of residual protons or carbon atoms of deuterated dimethyl sulfoxide.

$^{1}$H-NMR spectra of free (a) β-CD and (b) β-CD:Res (2:1) inclusion complex (25ºC) (spectrometer JNM-ECA Jeol 400 operating at 400 MHz (frequency 399.78)

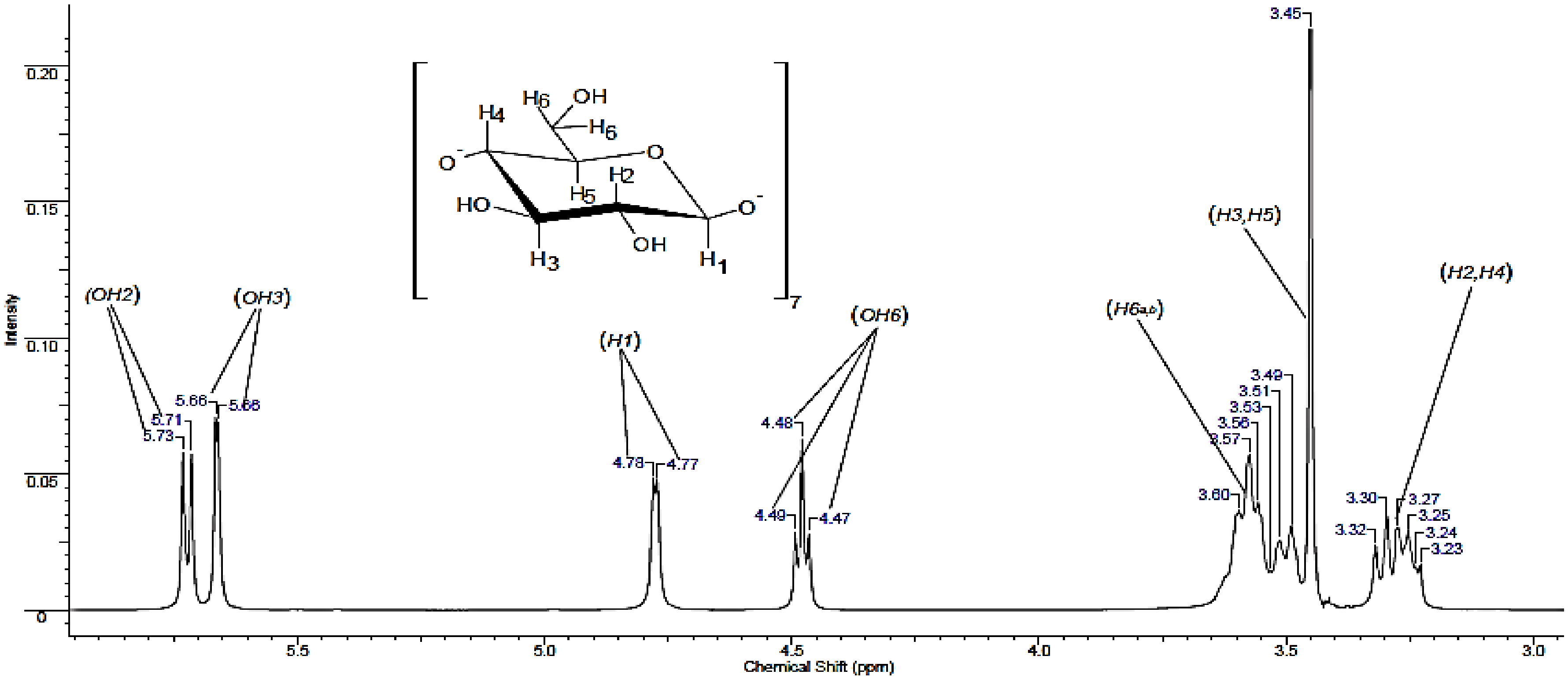


| No. | (ppm) | (Hz) | Height | No. | (ppm) | (Hz) | Height |
|---|---|---|---|---|---|---|---|
| 1 | 3.23 | 1290.5 | 0.0166 | 12 | 3.57 | 1428.8 | 0.0572 |
| 2 | 3.24 | 1294.1 | 0.0159 | 13 | 3.60 | 1437.5 | 0.0366 |
| 3 | 3.25 | 1300.5 | 0.0288 | 14 | 4.47 | 1785.0 | 0.0281 |
| 4 | 3.27 | 1308.8 | 0.0303 | 15 | 4.48 | 1790.5 | 0.0629 |
| 5 | 3.30 | 1317.9 | 0.0353 | 16 | 4.49 | 1796.0 | 0.0288 |
| 6 | 3.32 | 1327.1 | 0.0237 | 17 | 4.77 | 1907.8 | 0.0476 |
| 7 | 3.45 | 1379.8 | 1.0000 | 18 | 4.78 | 1911.0 | 0.0481 |
| 8 | 3.49 | 1394.9 | 0.0308 | 19 | 5.66 | 2262.7 | 0.0693 |
| 9 | 3.51 | 1404.5 | 0.0256 | 20 | 5.66 | 2264.5 | 0.0709 |
| 10 | 3.53 | 1412.3 | 0.0142 | 21 | 5.71 | 2284.2 | 0.0575 |
| 11 | 3.56 | 1421.9 | 0.0394 | 22 | 5.73 | 2291.1 | 0.0580 |

1H_b-CD in_DMSO-$d_6$

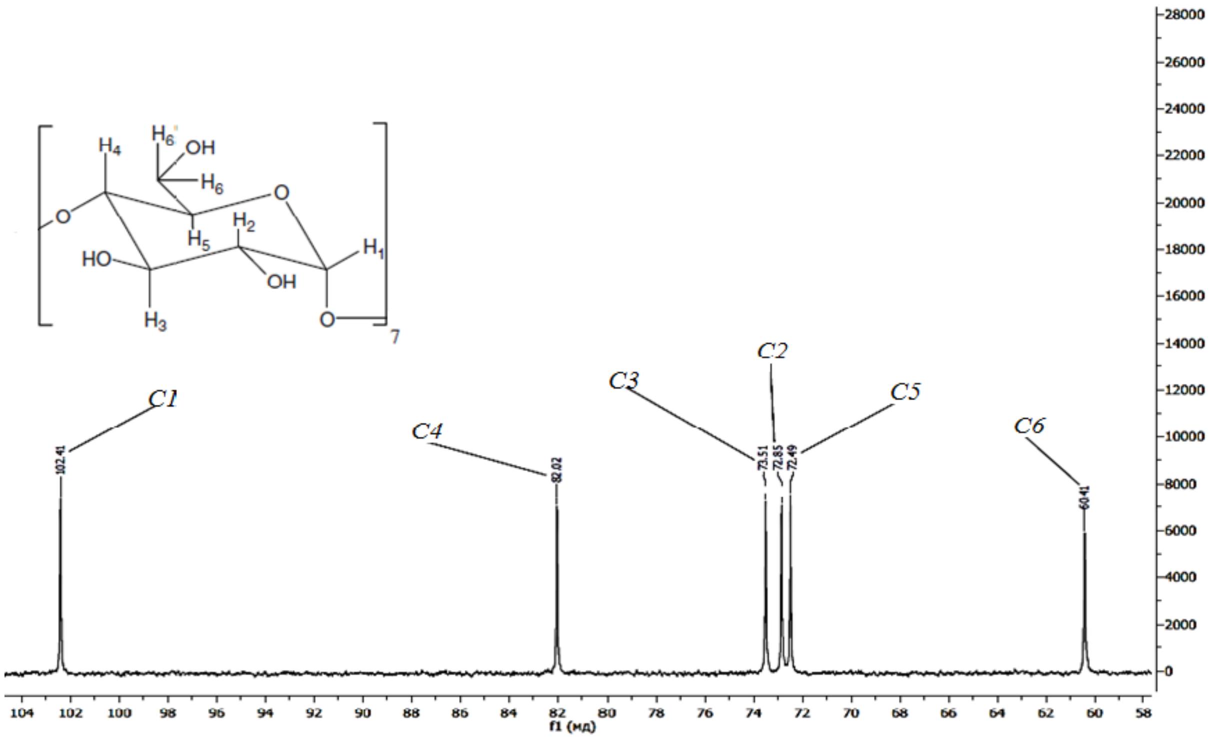


$^{13}C$ NMR spectra of free β-CD

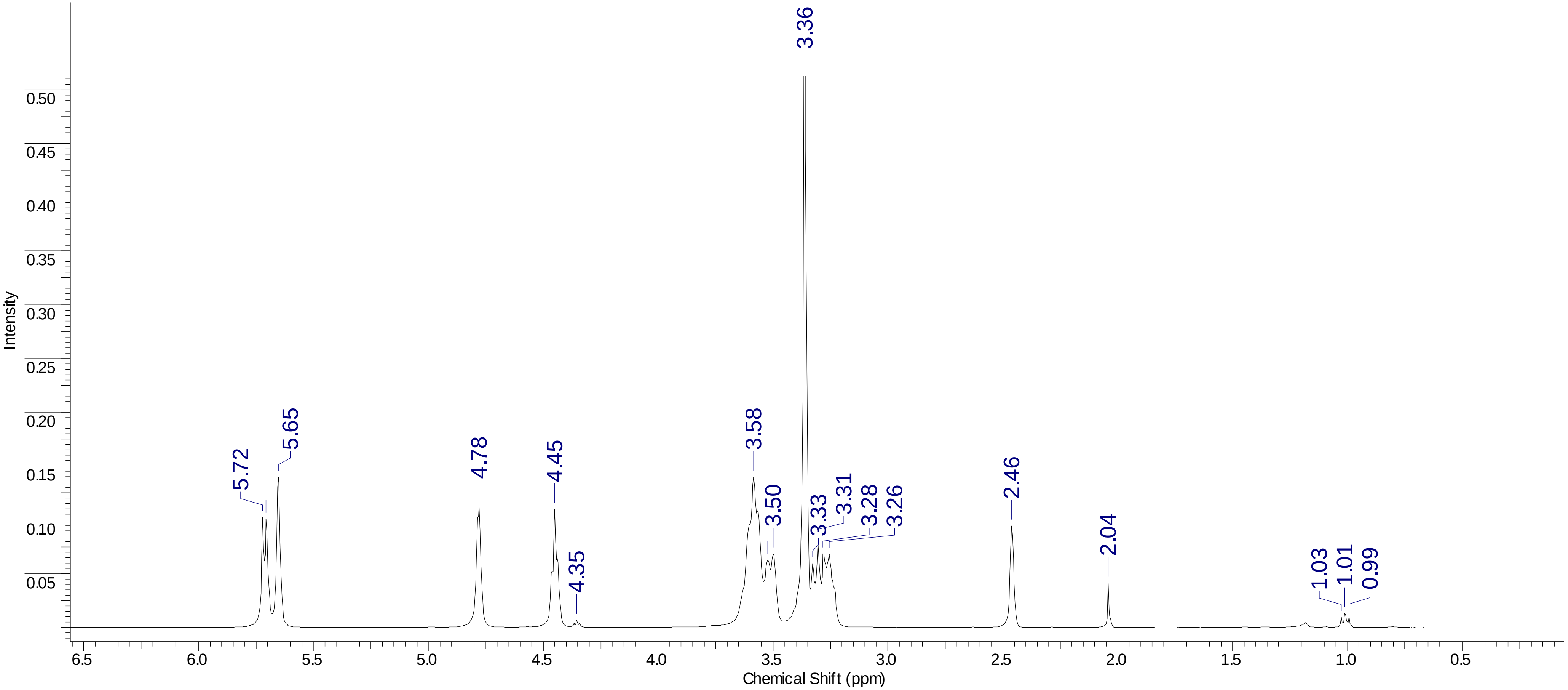


| No. | (ppm) | (Hz) | Height |
|---|---|---|---|
| 1 | 0.99 | 396.7 | 0.0098 |
| 2 | 1.01 | 404.4 | 0.0132 |
| 3 | 1.03 | 410.5 | 0.0097 |
| 4 | 2.04 | 816.4 | 0.0414 |
| 5 | 2.46 | 984.2 | 0.0942 |
| 6 | 3.26 | 1301.6 | 0.0681 |
| 7 | 3.28 | 1312.3 | 0.0683 |
| 8 | 3.31 | 1321.4 | 0.0795 |
| 9 | 3.33 | 1330.6 | 0.0595 |
| 10 | 3.36 | 1344.3 | 1.0000 |
| 11 | 3.50 | 1399.2 | 0.0684 |
| 12 | 3.52 | 1408.4 | 0.0628 |
| 13 | 3.58 | 1432.8 | 0.1397 |
| 14 | 4.35 | 1741.0 | 0.0067 |
| 15 | 4.45 | 1779.2 | 0.1096 |
| 16 | 4.78 | 1910.4 | 0.1131 |
| 17 | 5.65 | 2259.8 | 0.1398 |
| 18 | 5.71 | 2281.2 | 0.1007 |
| 19 | 5.72 | 2287.3 | 0.1019 |

1H_b-CD-resveratrol (2-1)_in_DMSO-$d_6$

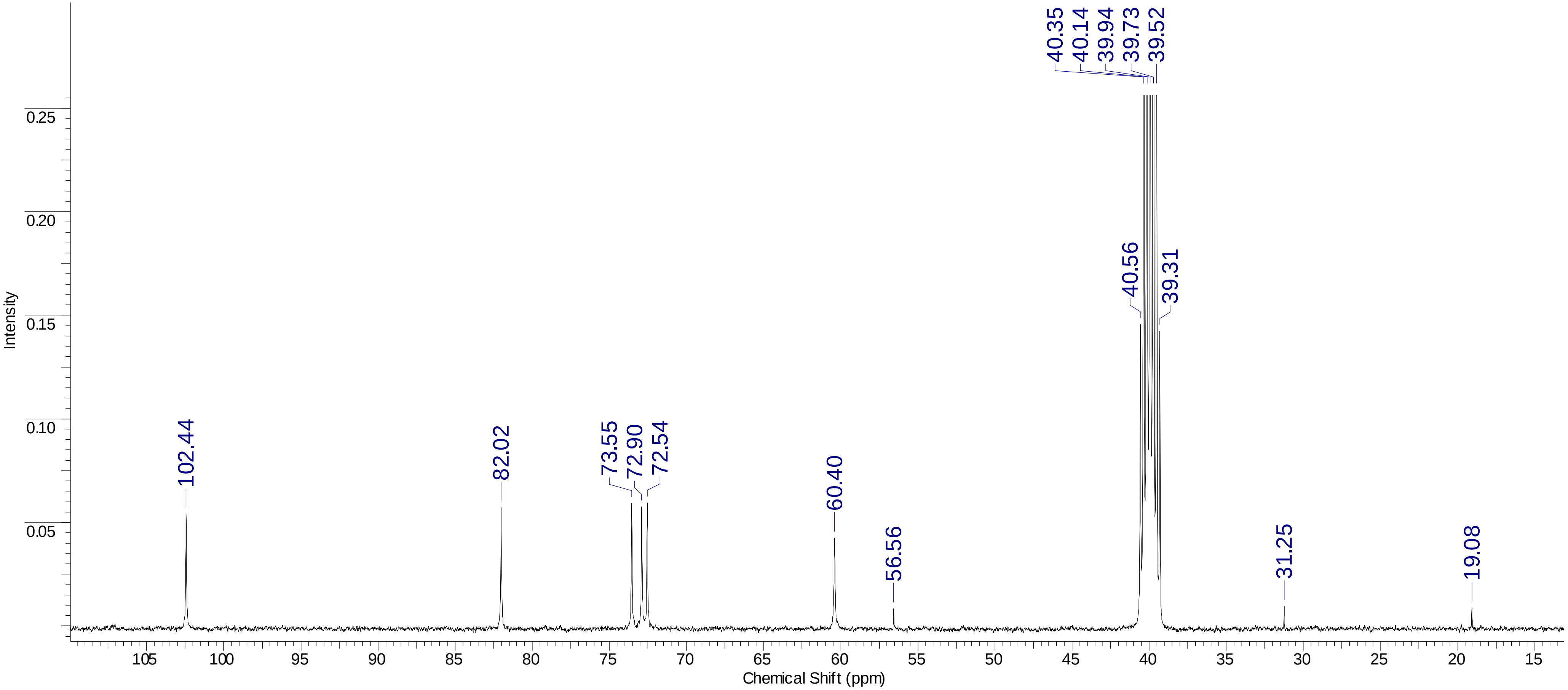


| No. | (ppm) | (Hz) | Height | No. | (ppm) | (Hz) | Height |
|---|---|---|---|---|---|---|---|
| 1 | 19.08 | 1918.0 | 0.0089 | 9 | 40.56 | 4077.4 | 0.1456 |
| 2 | 31.25 | 3141.0 | 0.0095 | 10 | 56.56 | 5685.8 | 0.0084 |
| 3 | 39.31 | 3951.9 | 0.1423 | 11 | 60.40 | 6072.0 | 0.0424 |
| 4 | 39.52 | 3973.0 | 0.4305 | 12 | 72.54 | 7292.1 | 0.0594 |
| 5 | 39.73 | 3994.1 | 0.8566 | 13 | 72.90 | 7328.6 | 0.0577 |
| 6 | 39.94 | 4015.1 | 1.0000 | 14 | 73.55 | 7393.7 | 0.0592 |
| 7 | 40.14 | 4035.3 | 0.8516 | 15 | 82.02 | 8244.9 | 0.0572 |
| 8 | 40.35 | 4056.4 | 0.4344 | 16 | 102.44 | 10297.9 | 0.0538 |

13C_b-CD-restver(2-1)_in_DMSO
